# Lentinan has a beneficial effect on cognitive deficits induced by chronic *Toxoplasma gondii* infection in mice

**DOI:** 10.1186/s13071-023-06023-5

**Published:** 2023-12-13

**Authors:** Shuxi Liu, Ziyi Yan, Yuan Peng, Yunqiu Liu, Yiling Li, Daxiang Xu, Yuying Gong, Zeyu Cui, Yongshui Wu, Yumei Zhang, Dahui Wang, Wei Pan, Xiaoying Yang

**Affiliations:** 1 Jiangsu Key Laboratory of Immunity and Metabolism, Department of Pathogen Biology and Immunology, Jiangsu International Laboratory of Immunity and Metabolism, Xuzhou Medical University, Xuzhou, 221004 Jiangsu China; 2 The First Clinical Medical College, Xuzhou Medical University, Xuzhou, Jiangsu China; 3 National Experimental Demonstration Center for Basic Medicine Education, Xuzhou Medical University, Xuzhou, Jiangsu China; 4 Department of Pharmacy, Affiliated Hospital of North Sichuan Medical College, Nanchong, Sichuan, China; 5 The Second Clinical Medical College, Xuzhou Medical University, Xuzhou, 221004 Jiangsu China; 6 Department of Pathogenic Biology, Binzhou Medical University, Binzhou, 256603 Shandong China; 7 Liangshan College (Li Shui) China, Lishui University, Lishui, 323000 Zhejiang China

**Keywords:** Lentinan, *Toxoplasma gondii*, Hippocampus, Cognitive deficits, Neuroinflammation

## Abstract

**Background:**

*Toxoplasma gondii* (*T. gondii*) is increasingly considered a risk factor for neurodegenerative diseases. However, there is only limited information on the development of drugs for *T. gondii* infection. Lentinan from *Lentinula edodes* is a bioactive ingredient with the potential to enhance anti-infective immunity. The present study aimed to investigate the neuroprotective effect of lentinan on *T. gondii*-associated cognitive deficits in mice.

**Methods:**

A chronic *T. gondii* infection mouse model was established by administering 10 cysts of *T. gondii* by gavage. Lentinan was intraperitoneally administered 2 weeks before infection. Behavioral tests, RNA sequencing, immunofluorescence, transmission electron microscopy and Golgi-Cox staining were performed to assess the effect of lentinan on cognitive deficits and neuropathology in vivo. In vitro, the direct and indirect effects of lentinan on the proliferation of *T. gondii* tachyzoites were evaluated in the absence and presence of BV-2 cells, respectively.

**Results:**

Lentinan prevented *T. gondii*-induced cognitive deficits and altered the transcriptome profile of genes related to neuroinflammation, microglial activation, synaptic function, neural development and cognitive behavior in the hippocampus of infected mice. Moreover, lentinan reduced the infection-induced accumulation of microglia and downregulated the mRNA expression of proinflammatory cytokines. In addition, the neurite and synaptic ultrastructural damage in the hippocampal CA1 region due to infection was ameliorated by lentinan administration. Lentinan decreased the cyst burden in the brains of infected mice, which was correlated with behavioral performance. In line with this finding, lentinan could significantly inhibit the proliferation of *T. gondii* tachyzoites in the microglial cell line BV2, although lentinan had no direct inhibitory effect on parasite growth.

**Conclusions:**

Lentinan prevents cognitive deficits via the improvement of neurite impairment and synaptic loss induced by *T. gondii* infection, which may be associated with decreased cyst burden in the brain. Overall, our findings indicate that lentinan can ameliorate *T. gondii*-related neurodegenerative diseases.

**Graphical Abstract:**

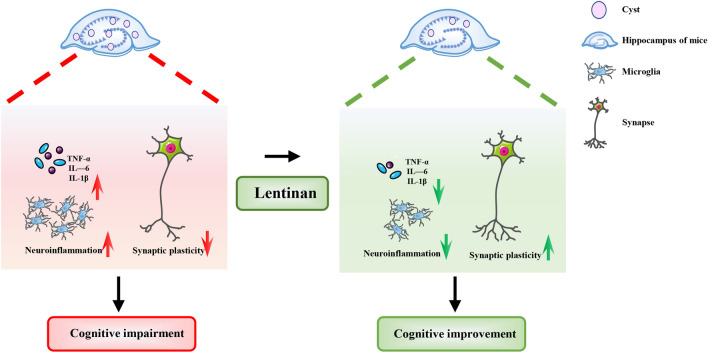

**Supplementary Information:**

The online version contains supplementary material available at 10.1186/s13071-023-06023-5.

## Background

Cognitive deficits, characterized by memory loss and declines in balance control, executive function and attention, are prevalent with an increasing incidence in the elderly population [1]. Unfortunately, current therapies and prevention strategies that are effective in the treatment and prevention of cognitive deficits are limited [2]. *Toxoplasma gondii* is a widespread zoonotic and neurotropic protozoan that has infected up to one third of the global population [3]. This parasite can penetrate the blood-brain barrier and form cysts in the brain, resulting in chronic, lifelong and latent infection [4]. In recent years, persistent exposure to *T. gondii* has been recognized as a vital risk factor for neurodegenerative disorders, including Alzheimer's disease (AD) [5]. Moreover, higher anti-*T. gondii* antibody levels were observed in the serum of AD patients compared with healthy controls [6]. Studies in animal models have shown that chronic *T. gondii* infection reduces nerve fiber density, impairs synapses and induces neuroinflammation [7], all hallmarks of neurodegenerative disease. Accordingly, these animals exhibited cognitive deficits, including disrupted memory and learning and altered social novelty recognition [8].

The current first-line therapy for toxoplasmosis includes dihydrofolate reductase inhibitors (pyrimethamine or trimethoprime) and dihydropteroate synthetase inhibitors (sulfadiazine, sulfadoxine or sulfamethoxazole) [9]. Moreover, clindamycin and atovaquone have been adopted as second-line therapy [10]. Unfortunately, these clinical drugs are only effective in eliminating tachyzoites in the acute stage and are unable to kill cysts in the chronic stage [11]. This inability to kill cysts may be because these drugs cannot pass the blood-brain barrier. Furthermore, several studies have shown that cyst number is associated with behavioral changes in *T. gondii*-infected mice [12, 13]. Thus, decreasing the cyst burden in the brain may have a beneficial effect on the cognitive deficits induced by parasitic infection.

Neuroinflammation is an important pathophysiological feature in neurodegenerative diseases [14, 15]. As the main resident immune cells of the central nervous system (CNS), microglia maintain brain homeostasis and contribute to brain development during normal conditions [16]. In disease conditions, activated microglia can release numerous proinflammatory factors, such as tumor necrosis factor-α (TNF-α), interleukin-6 (IL-6) and IL-1β, which can directly induce neuronal apoptosis [17]. In addition, activated microglia can directly engulf synapses, leading to synaptic loss [18–20]. Notably, numerous studies have shown that chronic *T. gondii* infection induces microglial activation, which is closely connected to neuronal damage and abnormal behaviors [21–23]. Recently, a study reported that inhibition of neuroinflammation reversed hyperactivity induced by chronic *T. gondii* infection [24]. Therefore, targeting the regulation of neuroinflammation may be an effective strategy for treating cognitive impairment induced by *T. gondii* infection.

*Lentinula edodes* is a widely cultivated edible and medicinal mushroom with multiple beneficial health effects [25]. Lentinan is a major biologically active polysaccharide extracted from *L. edodes* that exerts an immunostimulatory effect and has been clinically used for treating cancers and other diseases (e.g. hepatitis and HIV) [26–28]. Interestingly, several recent studies have reported that lentinan can resist infection by *Trichinella spiralis*, murine malaria and *Leishmania donovani* [29–31]. For example, lentinan can trigger the removal of *Trichinella spiralis* by modulating intestinal dysbiosis in mice [29]. Combined with miltefosine, lentinan can decrease *Leishmania donovani* infection by enhancing the phagocytic activity of macrophages [30]. Similar to *T. gondii*, murine malaria belongs to the Apicomplexan [32]. Lentinan can prevent the propagation of murine malaria by activating the protective Th1 immune response [31]. Our recent study found that mushroom-derived β-glucan can ameliorate anxiety‑like behavior by decreasing cysts in mice chronically infected with *T. gondii* [13]. Additionally, we also reported that dietary lentinan alleviates cognitive impairment in high-fat diet (HFD)-induced obese mice [33]. Another study showed that lentinan treatment improved depression-like behavior in mice [34]. These findings suggested that lentinan may exert an antiparasitic and neuroprotective effect. Thus, we hypothesized that lentinan may prevent cognitive impairment induced by chronic *T. gondii* infection.

In the present study, using a chronic *T. gondii* infection mouse model established by administering cysts of the *T. gondii* strain TgCtwh6, a low virulence strain dominantly circulating in China, by gavage [13], we evaluated the effect of lentinan on cognitive deficits, neuroinflammation and neuronal pathology. Furthermore, we evaluated the direct and indirect antiparasitic effects of lentinan in vitro by cultivating tachyzoites of the *T. gondii* RH strain [35] in the absence or presence of microglial BV-2 cells. We demonstrated that lentinan attenuates cognitive impairment induced by chronic *T. gondii* infection in mice. Moreover, lentinan decreased the cyst number of *T. gondii*, suppressed microglial activation, downregulated proinflammatory cytokine expression and alleviated neurite degeneration and synaptic loss in the hippocampus of mice. In vitro, lentinan could not directly inhibit the proliferation of *T. gondii* tachyzoites but could decrease the tachyzoite burden in BV2 cells. These data suggested that lentinan may be a potential drug candidate for treating *T. gondii*-related cognitive deficits.

## Methods

### Ethics approval

All animal care and experiments were followed the guidelines for laboratory animal care and use of the National Institutes of Health and were approved by the Ethics Committee (LAWEC) of Xuzhou Medical University (Xuzhou, China, SCXK (Su) 2020-0048).

### Parasite preparation

TgCtwh6 strain was acquired from Prof. Jilong Shen’s laboratory. The cysts of TgCtwh6 were isolated from brain tissues of TgCtwh6-infected mice (4 weeks after infection), resuspended in 1 ml PBS and then counted microscopically.

### Mouse model of *T. gondii* infection and lentinan treatment

Seven-week-old C57BL/6J mice were provided by the Experimental Animal Center of Xuzhou Medical University (Xuzhou, China) and were housed in environmentally controlled conditions (temperature 22-24 °C, free access to water and food, and 12 h light/dark cycle). Mice were randomly assigned to four groups (*n* = 10/group, 5 male mice and 5 female mice): (i) mice intraperitoneally injected with PBS and received PBS by gavage 2 weeks later as a vehicle control (Con) group; (ii) mice intraperitoneally injected with lentinan (50 mg/kg/mouse, Yuanye Biological Technology Co., Ltd., Shanghai, China) and received PBS by gavage 2 weeks later as the (ConL) group; (iii) mice intraperitoneally injected with PBS that received ten cysts of TgCtwh6 by gavage 2 weeks later as the *T. gondii*-infected (Tg) group [36]; (iv) mice intraperitoneally injected with lentinan (50 mg/kg/mouse) and received *T. gondii* infection 2 weeks later as the lentinan-treated (TgL) group. 4 weeks post infection, behavioral tests were performed. Then, mice were killed with CO_2_, and brain tissues were immediately collected for further analyses.

### Nest building test

The nest building test was performed according to the previous procedure [37]. Briefly, mice were housed individually in a cage with unrestricted water and food. About 1 h before the dark stage, one nestlet weighing 3 g was placed in each cage. The deacon nest score and untore nestlet weight were evaluated the next morning to assess the activities of typical daily living according to a previously reported method [38].

### Objection location test (OL) and novel object recognition test (NOR)

The OL and NOR tests were carried out as previously described [39]. Briefly, the day before testing, mice were placed into the testing box to explore the box freely for 5 min. In the exploration phase of the testing day, mice were presented with two identical objects placed parallelly and allowed to explore the box freely for 5 min. Then, the retention session took place for 1 h. In the recognition phase of OL, one of the objects was transferred to the diagonal place, and mice were allowed to explore the box freely for 5 min. The place discrimination index (PDI) was calculated by using the formula: (the time spent with the object moved to a novel place/the total time spent in exploring both objects) × 100. In the recognition phase of NOR, one of the objects was replaced by a novel object, and mice were allowed to explore the box with a familiar object and a novel object placed parallelly for 5 min. The novel object discrimination index (NODI) was evaluated by using the formula: (the time spent in exploring novel object/the total time spent in exploring both objects) × 100.

### Cyst burden assay

The cyst burden of the brain was evaluated based on a previous protocol [40]. Briefly, brains obtained from Tg and TgL mice were mechanically homogenized in 1 ml PBS and the cyst number was counted under the light microscopy (20×).

### Dendritic spine morphology assay

The variations of dendritic spine morphology were analyzed as previously described [41]. Briefly, the whole brains of mice were quickly dissected, rinsed in PBS and impregnated with the solutions of the FD Rapid Golgi Stain™ Kit (#PK401, FD NeuroTechnologies, Inc., Columbia, MD, USA) according to the manufacturer’s instructions. Then, brain tissue was sectioned to 200 μm using a vibratome (Leica, Wetzlar, Germany), mounted on gelatin-coated slides and stained with solutions provided in the kit. Finally, slices were dehydrated with alcohol in increasing concentrations, cleared in xylene and mounted with neutral balsam (G8590, Solarbio, Beijing, China). Imaging was conducted using an Olympus XM10 microscopy (Olympus, Richmond Hill, ON, Canada) equipped with a CCD camera. The pyramidal neurons from the hippocampal CA1 region were randomly selected for analysis. The dendritic tracings of neurons were quantified by Sholl analysis as described elsewhere [42] to evaluate the total neurite length of the neuron, neurite length of branches, number of neurite branches per neuron and intersections. The spine density of dendritic spines along 10 μm of a distal branch was counted as previously reported [43].

### Tachyzoite survival test

Several studies have successfully investigated the viability of *T. gondii* tachyzoites in vitro [44–46]. In the present study, the tachyzoite survival test was carried out as previously described [44]. Briefly, to evaluate the direct inhibition effect of lentinan against *T. gondii*, free tachyzoites from *T. gondii* RH strain (a highly virulent strain of *T. gondii* acquired from Prof. Guorong Yin’s laboratory) were cultivated in vitro with 0.8, 4, 20, 100 and 250 μg/ml lentinan (Yuanye Biological Technology Co., Ltd, Shanghai, China) or 25, 50, 100, 200 and 400 μg/ml sulfadiazine/SD (Bolida Technology Co., Ltd, Xuzhou, China) at a density of 1 × 10^5^/well in a 96-well microplate for 36 h. Then, the tachyzoites were collected and stained with trypan blue. The numbers of stained tachyzoites were counted using an inverted fluorescence microscope (*IX51, Olympus, Japan). The inhibition rate of *T. gondii* tachyzoite = [tachyzoite numbers in culture medium with dimethy1 sulfoxide (DMSO) − tachyzoite numbers in medium with the indicated concentration of lentinan or SD]/tachyzoite numbers in culture medium with DMSO × 100%], according to the literature [44].

### Cell culture and *T. gondii* infection

The murine microglial cell line BV2 cells were obtained from Shanghai Cell Research Center (Shanghai, China) and were cultured in Dulbecco’s modified Eagle’s medium (DMEM) containing 4.5 g/l glucose, 10% FBS and 1% penicillin/streptomycin with 5% CO_2_ at 37 °C. For the prevention experiment, BV2 cells were seeded at a density of 5 × 10^4^/well in 12-well plates until confluency and then stimulated with 0.8, 4, 20, 100 and 250 μg/ml lentinan or 100 μg/ml SD for 5 h. After removing the culture medium, BV2 cells were cultured in a medium containing 2.5 × 10^5^ tachyzoites of *T. gondii* RH strain/well for 4 h. Then, *T. gondii*-infected BV2 cells were washed with medium and incubated for another 36 h. For the therapeutic experiment, BV2 cells were infected with 2.5 × 10^5^ tachyzoites/well for 4 h. After removing extracellular parasites, BV2 cells were stimulated with indicated concentrations of lentinan or SD for 36 h.

### Immunofluorescence

For image analysis of hippocampal immunofluorescence, sections were processed as previously described [47]. The dissected brains were paraformaldehyde fixed and then dehydrated in PBS containing 30% sucrose the following day and preserved at − 20 °C freezer. Sections were cut into 20 μm thicknesses using a rotary microtome (RM2016, Leica, German). For double immunofluorescence staining, the brain sections were incubated with the rabbit anti-interleukin-6 (IL-6) antibody (gb11117, 1:200, Servicebio, China) for the remainder of the day at 4 °C after blocking with BSA (G5001, Servicebio) for 30 min. Next, the brain sections were incubated for 50 min with HRP-linked goat anti-rabbit IgG secondary antibody (gb21303, 1:300, Servicebio), washed with TBST buffer and then incubated with FITC-TSA (G1222, 1:1000, Servicebio) for 10 min. Following washing with TBST buffer, the sections were heated in an EDTA antigen repair buffer-filled repair box (G1206, Servicebio) to remove the bound antibodies and incubated with the anti-calcium-binding adapter molecule 1 (Iba-1, Ab178847, 1:100, Abcam) at 4 °C overnight. Then, sections were washed and incubated with Cy3 conjugated goat anti-rabbit IgG secondary antibody (gb21303, 1:300, Servicebio) for 50 min. For immunofluorescence staining with the Iba-1 antibody, the brain sections were incubated with the anti-Iba-1 antibody at 4 °C overnight after blocking with BSA (G5001, Servicebio) for 30 min at ambient temperature. Then, sections were washed and incubated with Cy3 conjugated goat anti-rabbit IgG secondary antibody (gb21303, 1:300, Servicebio) for 50 min at ambient temperature. Finally, the slices were stained with DAPI (G1012, Servicebio). Photographs were captured in the CA1, CA3 and DG regions of the hippocampus using a fluorescence microscope (Eclipse C1, Nikon, Japan). The number, morphology and the IL-6^+^ cell percentage of Iba1^+^ microglia cells and the mean fluorescence intensity of IL-6^+^ cells were quantified using Image J software. For cell morphology analysis, the circularity and solidity of Iba1^+^ cells were quantified using methods described previously [47].

For immunofluorescence staining with anti-*T. gondii* antibody, BV2 cells were fixed with pre-cooled 4% paraformaldehyde for 15 min, permeabilized with 0.2% TritonX-100 in PBS for 15 min and incubated with PBS containing 2% BSA for 1.5 h. Then, BV2 cells were incubated with the primary antibody against *T. gondii* (ab138698, 1:1000, Abcam) 150 μl/well at 4 °C overnight. After washing with PBS, the cells were incubated with Alexa Fluor® 488-conjugated goat anti-Mouse IgG (H+L) secondary antibody (gb25301, 1:300, Servicebio) for 1 h. Finally, the cells were stained with DAPI ( G1012, Servicebio). The images were captured using a fluorescence microscope (Eclipse C1, Nikon, Japan). *T. gondii* tachyzoites were shown colored in green and the nuclei in blue [48, 49].

### Transmission electron microscopy (TEM)

Mice were transcardially perfused with saline after being killed. The brains were quickly taken out, and the hippocampus CA1 region was dissected and rapidly fixed in a mixture of 2% paraformaldehyde-2.5% glutaraldehyde for 24 h. Then, the steps of sample preparations before TEM observation were processed according to the method described previously [47]. Synaptic morphometry was viewed on a transmission electron microscope (HT7800, Hitachi, Japan), and asymmetric synapses were identified in the micrographs. The postsynaptic density (PSD) thickness (the distance along a perpendicular line traced from the postsynaptic membrane to the area of the synaptic complex that is most convex), width of the synaptic clefts (SC) (the widest and narrowest parts of the synapse and averaged these values), length of the active zone (AC) and synaptic curvature (the ration of synaptic post interface arch length and chord length, which is closely related to neurotransmitter efficiency [50, 51]) from 10 synapses among groups were estimated as described in the previous study [37].

### RNA sequencing

Total RNA from the fresh hippocampus of mice was extracted using TRIzol^®^ reagent kit (Invitrogen, Carlsbad, CA, USA) to analyze transcriptome profile changes. The mRNA was enriched by oligo (dT) beads and reverse-transcribed into cDNA with random primers. DNA polymerase I, RNase H, dNTP and buffer were used to synthesize second-strand cDNA. After that, the cDNA fragments were purified with a QiaQuick PCR extraction kit and end-repaired,  added poly(A), and then ligated to Illumina sequencing adapters. Ligated fragments were subjected to agarose gel electrophoresis for size selection, PCR amplification and cDNA library preparation. The resulting cDNA library was sequenced using Illumina HiSeq 2500 by Gene Denovo Biotechnology Co. (Guangzhou, China). The edge R package (http://www.r-project.org) was used to identify differentially expressed genes (DEGs) across groups. DEGs with |log_2_(fold change)|  ≥ 1 or ≤ − 1 and *P* < 0.05 were considered significantly modulated [52]. The Gene Ontology (GO) enrichment analysis was performed using the DAVID Bioinformatics Resources 6.8 (https://david.ncifcrf.gov/) to identify the potential biological functions. The Kyoto Encyclopedia of Genes and Genomes (KEGG) pathway analysis was conducted to predict the possible signal pathways by using the database (http://www.kegg.jp/). The STRING database (https://stringdb.org/, v.10.5) and Cytoscape 3.9.1 were used to perform the PPI network.

### Quantitative real-time PCR(qRT-PCR)

Total RNA was extracted from BV2 cells and the hippocampus of mice. The PCR method was performed as previously reported [37]. The relative mRNA expression level was determined using the formula 2^−ΔΔCt^ and β-actin as the internal reference control. All primers are listed in Additional file 1: Table S1.

### Statistical analysis

All data are shown as the mean ± standard error of mean (SEM). Statistical analysis was performed using the GraphPad Prism 8.0 software (GraphPad Software, San Diego, CA, USA). After data were tested for normality, the differences between two groups were determined by using Student’s *t*-test, while differences among three or more groups were evaluated by using one-way analysis of variance (ANOVA) followed by the post hoc Tukey-Kramer test. *P* < 0.05 was considered statistical significance.

## Results

### Lentinan ameliorated cognitive deficits in chronic *T. gondii*-infected mice

**Fig. 1:**
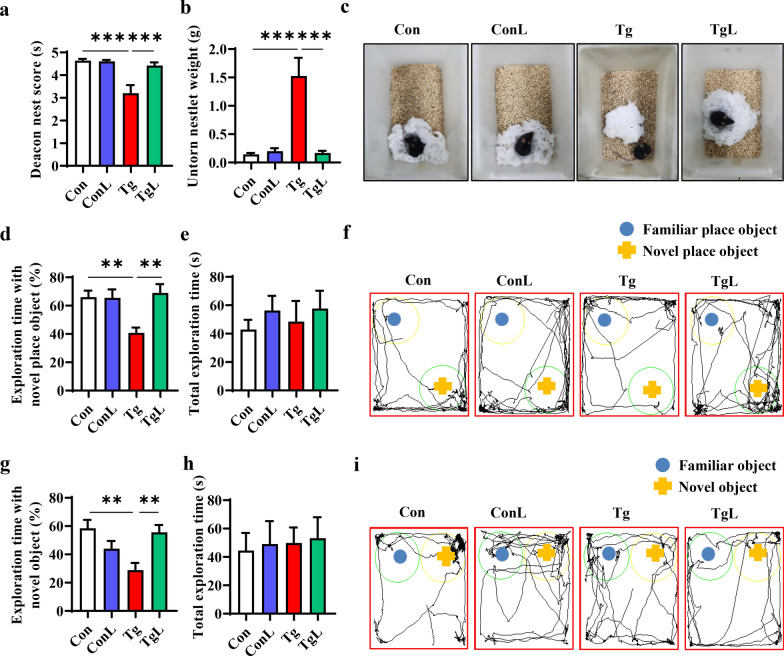
Lentinan ameliorated cognitive deficits in chronic *Toxoplasma gondii*-infected mice. The nest building test was used to estimate the ability of daily living of mice (**a**–**c**). **a** The nest score. **b** Untore nestler weight (amount of untore nesting material). **c** Representative nest of Con, ConL, Tg and TgL mice. The object location test was performed to evaluate the function of spatial memory of the mice (**d**–**f**). **d** Percentage of exploration time spent with the object in the novel place to total object exploration time. **e** The total object exploration time spent with the object in the familiar and novel place. **f** Representative track plots of Con, ConL, Tg and TgL mice recorded by SMART video tracking system in the testing phase. The novel object recognition test was performed to evaluate the object recognition memory of the mice (**g**–**i**). **g** Percentage of exploration time spent with the novel object to total object exploration time. **h** The total object exploration time. **i** Representative track plots of Con, ConL, Tg and TgL mice. Con: control group (*n* = 10); ConL: Con + Lentinan group (*n* = 10); Tg: chronic *T. gondii*-infected group (*n* = 10); TgL: Tg + Lentinan group (*n* = 10). Values are mean ± SEM. **P < 0.01, ***P < 0.001

We first tested whether lentinan could improve toxoplasmosis-induced cognitive deficits with model mice infected with *T. gondii* TgCtwh6 cysts for 4 weeks [53]. Nest building, object location and novel object recognition tests were performed to assess the impact of lentinan administration on cognitive behaviors, including activities of daily living, spatial memory and recognition memory [38, 39]. The nest building test showed that *T. gondii*-infected mice, compared with the control mice, exhibited impaired activities of daily living consisting of a lower deacon nest score (the ability to build a nest) and a higher untorn nestlet weight (the nest-building deficit), while lentinan treatment significantly increased the deacon nest score and decreased the untorn nestlet weight (*F*_(3, 32)_ = 5.269, *P* < 0.001, Fig. 1a; *F*_(3, 30)_ = 12.35, *P* < 0.001, Fig. 1b, c). The object location test showed that lentinan induced a protective effect on spatial memory in *T. gondii*-infected mice, represented by an increase in the place discrimination index (PDI, percentage of time spent exploring an object in a novel place) (*F*_(3, 26)_ = 1.027, *P* < 0.01, Fig. 1d), while lentinan had no effect on the total exploration time with objects (*F*_(3, 24)_ = 1.079, Fig. 1e, f), indicating that the protective effect of lentinan was not due to variable general activity. The novel object recognition test showed that lentinan prevented recognition memory impairment in *T. gondii*-infected mice, as demonstrated by an increase in the novel object discrimination index (NODI, percentage of time spent with the novel object) (*F*_(3, 30)_ = 0.1110, *P* < 0.01, Fig. 1g, i). Likewise, the total exploration time of objects was comparable between the two groups (*F*_(3, 37)_ = 0.6669, Fig. 1h). Intriguingly, lentinan decreased the number of cysts in the brains of *T. gondii*-infected mice (*t*_*(*7)_ = 2.836, *P* < 0.05, Additional file 1: Fig. S1a). Moreover, potential correlations between cognitive behaviors and cyst numbers were investigated and showed that the deacon nest score, exploration time with a novel place object and exploration time with a novel object had negative correlations with cyst numbers (*r* = − 0.8844, *P* < 0.0001, Additional file 1: Fig. S1b; *r* = − 0.8443, *P* < 0.0001, Additional file 1: Fig. S1d; *r* = − 0.8011, *P* < 0.001, Additional file 1: Fig. S1e), while the untorn nestlet weight had a positive correlation with cyst numbers (*r* = 0.9233, *P* < 0.0001, Additional file 1: Fig. S1c). Overall, lentinan ameliorated the impairment of cognitive function induced by chronic *T. gondii* infection, which was closely associated with the decrease in cyst burden.

### Lentinan downregulated genes associated with neuroinflammation and microglial activation induced by chronic *T. gondii* infection

**Fig. 2:**
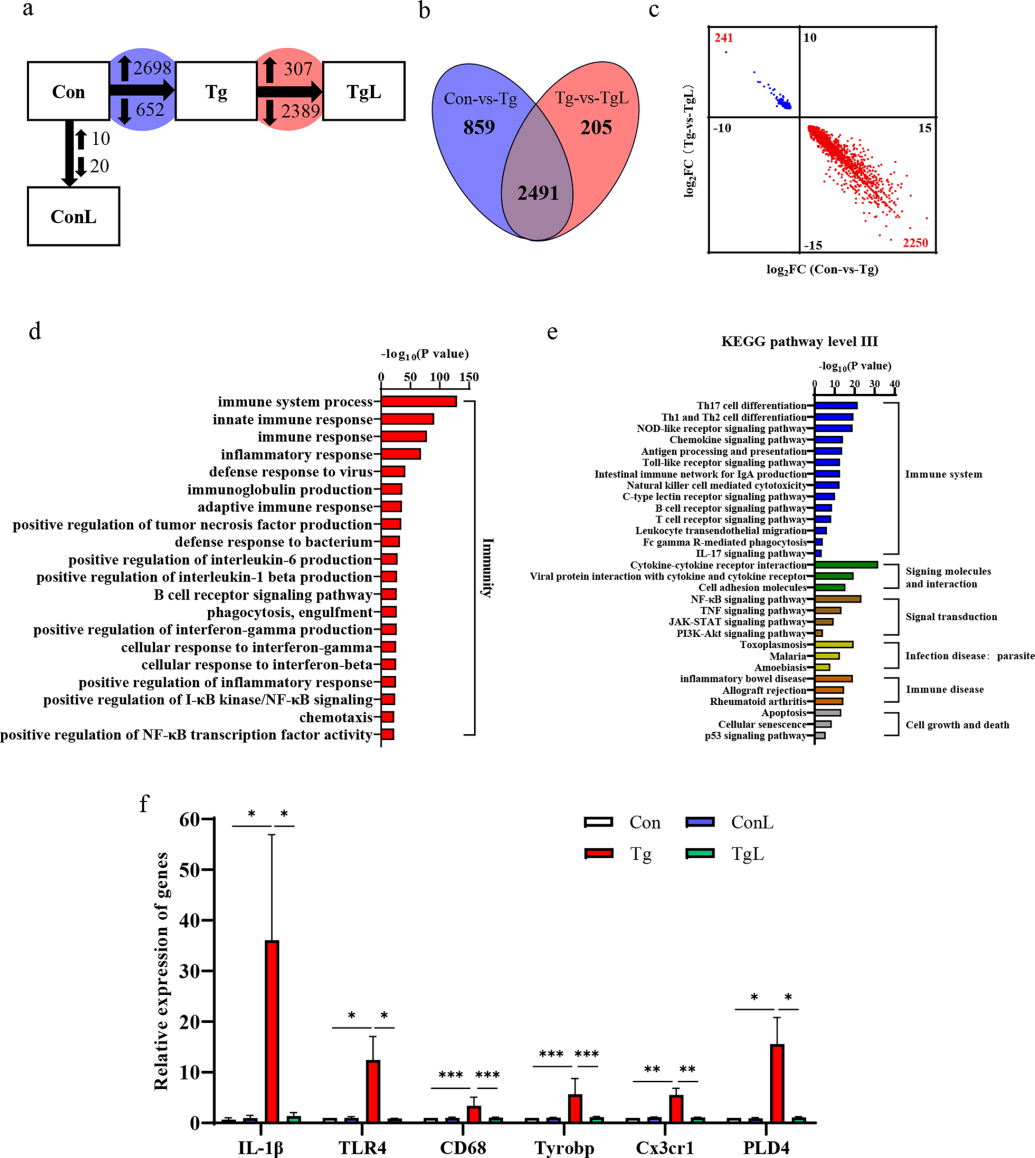
Lentinan suppressed the expression profile of genes associated with neuroinflammation and microglia activation induced by chronic *Toxoplasma gondii* infection. **a** Differential expression analysis revealed gene expression changes in the hippocampus of control (Con), ConL, Tg and TgL mice. Between Con and Tg mice (Blue Circle), 2698 genes were upregulated and 652 genes were downregulated; 2696 genes were changed between Tg and TgL mice (red circle), with 307 genes increased and 2389 genes decreased. **b** Venn diagram illustrating the overlap of 2491 genes that were changed in both Con vs. Tg (blue) and Tg vs. TgL (Red). **c** Scatter plot illustrating the 2491 overlapping genes showed log_2_FC(Con vs Tg, *x*-axis;) against log_2_FC(Tg vs TgL, *y*-axis;). The upper left quadrant represents 241 genes that had decreased expression with *T. gondii* infection and increased expression supplemented with lentinan. Conversely, the lower right quadrant illustrates 2250 genes that were increased with *T. gondii* infection and decreased with lentinan supplementation. **d** The top 20 enriched GO terms of biological process. **e** The enriched KEGG pathways. **f** Relative expression of IL-1β, TLR4, CD68, Tyrobp, Cx3cr1 and PLD4. Con: control group (*n* = 3); ConL: Con + Lentinan group (*n* = 3); Tg: chronic *T. gondii*-infected group (*n* = 3); TgL: Tg + Lentinan group (*n* = 3). Values are mean ± SEM. *P < 0.05, **P < 0.01, ***P < 0.001

To determine the effects of lentinan treatment on cognitive deficits at the transcriptome level, the hippocampus of Con, ConL, Tg and TgL mice was collected, and RNA sequencing (RNA-seq) was performed to identify the differentially expressed genes (DEGs). As shown in Fig. 2a, lentinan had a minimal impact on hippocampal gene expression in noninfected mice, as evidenced by 10 upregulated genes and 20 downregulated genes after lentinan administration. This result was consistent with the finding that cognitive behaviors were comparable between Con and ConL mice (Fig. 1). Compared with the Con mice, those infected with *T. gondii* exhibited a total of 3350 DEGs, including 2698 upregulated DEGs and 652 downregulated DEGs, while lentinan upregulated 307 DEGs and downregulated 2389 DEGs in the hippocampus of *T. gondii*-infected mice (Fig. 2a). Moreover, 2491 DEGs were regulated by both *T. gondii* infection and lentinan treatment, among which 2250 upregulated DEGs and 241 downregulated DEGs were found in the hippocampus of mice after *T. gondii* infection, while lentinan treatment reversed the expression of these DEGs (Fig. 2b). The detailed value of log_2_ (fold change) of 2491 DEGs is shown in Fig. 2c.

Gene Ontology (GO) analysis and Kyoto Encyclopedia of Genes and Genomes (KEGG) pathway analysis were carried out to identify the significantly enriched terms and signaling pathways in the 2250 DEGs that were upregulated by *T. gondii* infection and downregulated by lentinan treatment. The fold differential expression of the top 30 DEGs is listed in Additional file 1: Table S2. Interestingly, GO analysis showed that the top 20 enriched terms of the biological process were closely associated with immunity (Fig. 2d). These biological processes were related to the production of classic pro-inflammatory cytokines, such as “positive regulation of tumor necrosis factor production,” “positive regulation of interleukin-6 production” and “positive regulation of interleukin-1 beta production” (Fig. 2d). Moreover, KEGG pathway analysis also showed that pathways related to the proinflammatory response, including the Toll-like receptor signaling pathway, Fc gamma R-mediated phagocytosis, cytokine-cytokine receptor interaction, NF-κB signaling pathway and TNF signaling pathway, were significantly enriched (Fig. 2e). Consistent with the infection model, the pathway linked to “parasite infection disease: toxoplasmosis” was observed (Fig. 2e). Concomitantly, we found that *T. gondii* infection significantly upregulated the expression of genes involved in proinflammatory cytokine production and microglial activation (IL-1β, TLR4, CD68, Tyrobp, Cx3cr1 and PLD4), while lentinan abrogated these changes (IL-1β: *F*_(3, 8)_ = 2.558, *P* < 0.05; TLR4: *F*_(3, 8)_ = 1.913, *P* < 0.05; CD68: *F*_(3, 7)_ = 0.5318, *P* < 0.001; Tyrobp: *F*_(3, 7)_ = 83.91, *P* < 0.001; Cx3cr1: *F*_(3, 8)_ = 1.387, *P* < 0.01; PLD4: *F*_(3, 8)_ = 1.554, *P* < 0.05, Fig. 2f). Collectively, these transcriptome results suggested that lentinan supplementation substantially improved the abnormal hippocampal transcriptome related to neuroinflammation and microglial activation caused by chronic *T. gondii* infection.

### Lentinan altered the transcriptome profile of genes linked with cognitive function in the hippocampus of *T. gondii*-infected mice

**Fig. 3:**
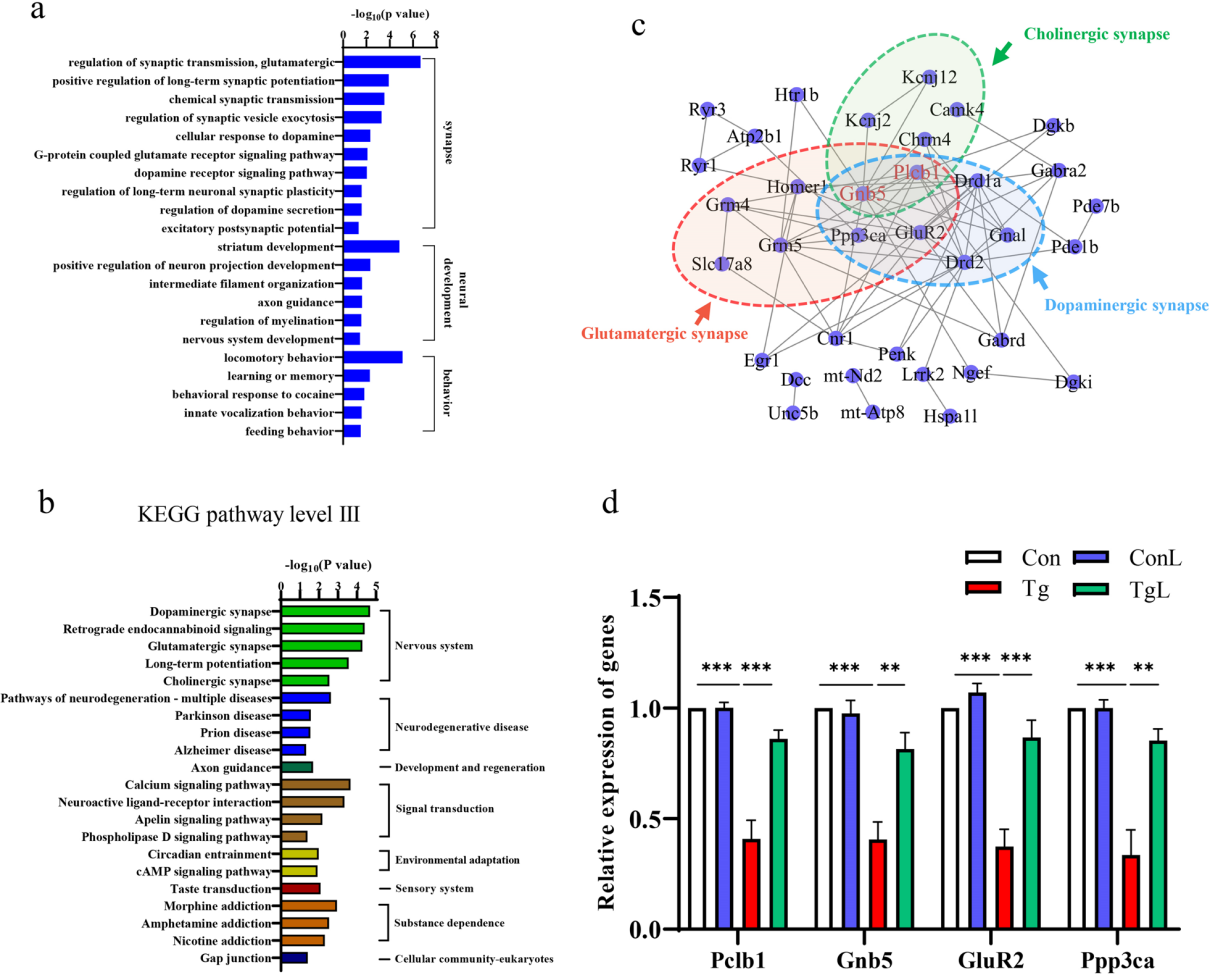
Lentinan altered the transcriptome profile of genes linked with cognitive function in the hippocampus of *Toxoplasma gondii*-infected mice. **a** Bar charts show the upregulated biological processes related to the synapse, neural development and behavior. **b** The enriched KEGG pathways. **c** PPI network analysis of genes from pathways in **b**. Black lines denoted the interaction between two genes. **d** Relative expression of Plcb1, Gnb5, GluR2, Ppp3ca. Con: control group (*n* = 3); ConL: Con + Lentinan group (*n* = 3); Tg: chronic *T. gondii*-infected group (*n* = 3); TgL: Tg + Lentinan group (*n* = 3). Values are mean ± SEM. **P < 0.01, ***P < 0.001

To uncover more transcriptome information about how lentinan improved cognitive deficits in *T. gondii*-infected mice, we further characterized the 241 DEGs that were downregulated by *T. gondii* infection and upregulated by lentinan treatment (Fig. 2c). For GO analysis, the total enriched biological processes are listed in Fig. 3a. Of these, 10 terms related to synaptic transmission, synaptic excitability and synaptic plasticity were significantly enriched (Fig. 3a). Moreover, six terms associated with neural development and five terms linked with behavior (learning or memory, feeding behavior, etc.) were identified (Fig. 3a). Detailed information on these DEGs related to synaptic function, neural development and behavior is listed in Additional file 1: Table S3. KEGG pathway analysis showed that pathways associated with synapse (dopaminergic synapse, glutamatergic synapse, etc.), neurodegenerative disease (PD, AD, etc.), development and regeneration (axon guidance), signal transduction (calcium signaling pathway, neuroactive ligand-receptor interaction, etc.), environmental adaptation (circadian entrainment and cAMP signaling pathway), sensory system (taste transduction) and substance dependence (morphine addiction, amphetamine addiction, etc.) were significantly enriched post-lentinan administration (Fig. 3b). Additionally, the PPI network was mapped to clarify the interaction of genes in the pathways listed in Fig. 3b and indicated that phosphoinositide phospholipase C-beta-1 (Plcb1) and guanine nucleotide-binding protein subunit beta-5 (Gnb5) were core nodes among dopaminergic synapse, glutamatergic synapse and cholinergic synapse. In addition, glutamate receptor 2 (GluR2) and protein phosphatase 3 catalytic subunit alpha (Ppp3ca) were core nodes between dopaminergic synapse and glutamatergic synapse (Fig. 3c). Moreover, we noticed that the abnormal expression of Plcb1, Gnb5, GluR2 and Ppp3ca in *T. gondii*-infected mice was significantly reversed with lentinan treatment (Plcb1: *F*_(3, 8)_ = 0.8883, *P* < 0.001; Gnb5: *F*_(3, 8)_ = 0.7755, *P* < 0.01; GluR2: *F*_(3, 8)_ = 0.2915, *P* < 0.001; Ppp3ca: *F*_(3, 8)_ = 1.763, *P* < 0.01, Fig. 3d). Overall, these transcriptome results indicated that lentinan restored the transcriptome profile linked with cognition in the hippocampus of *T. gondii*-infected mice, further supporting the role of lentinan in cognitive improvement.

### Lentinan ameliorated microglia activation triggered by *T. gondii* infection in the hippocampus

**Fig. 4:**
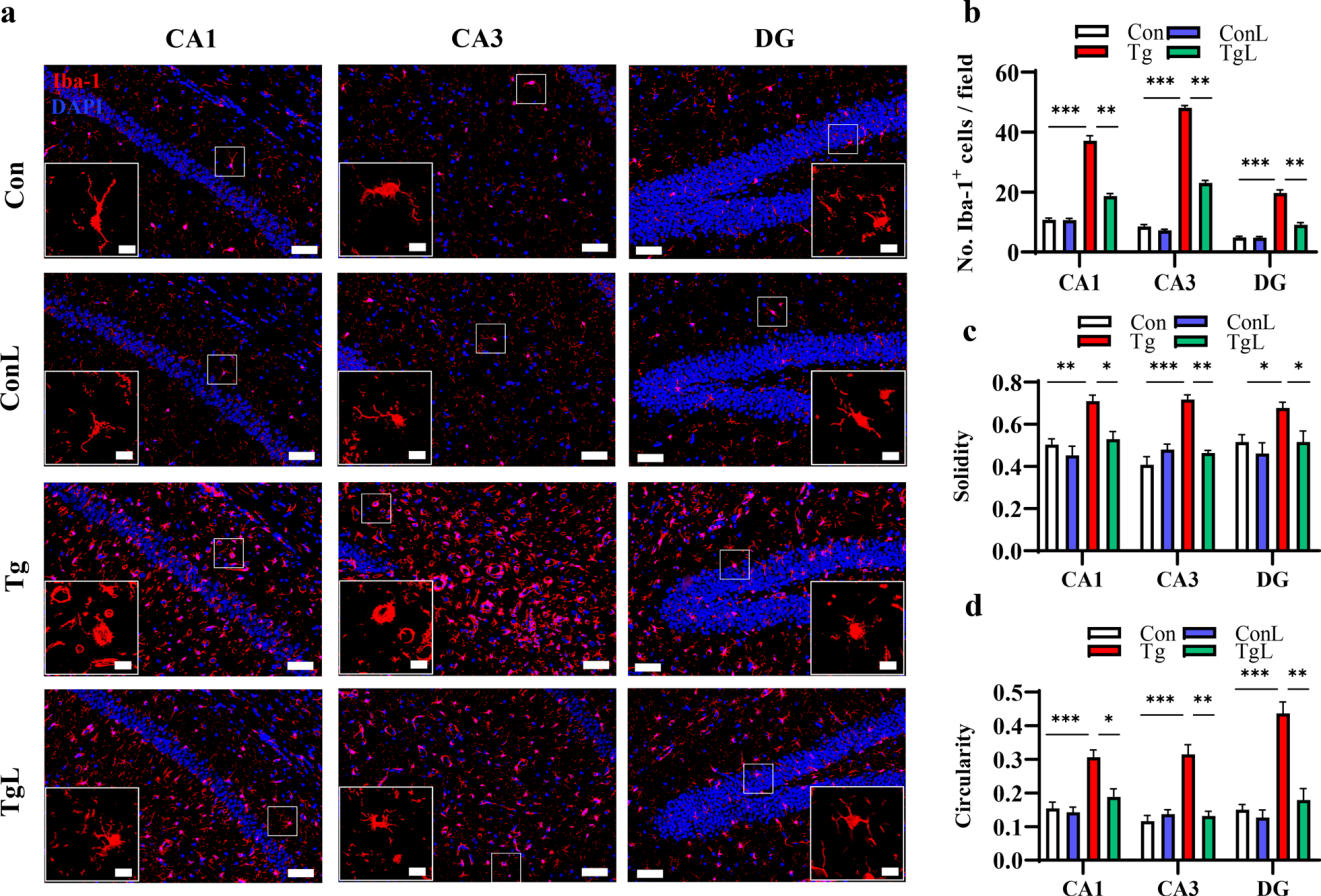
Lentinan ameliorated microglia activation triggered by *Toxoplasma gondii* infection in the hippocampus. **a** The immunofluorescent staining of Iba1 in CA1, CA3 and DG regions of the hippocampus (scale bar: 50 μm). The image captured from the box was marked with a dotted line (scale bar: 10 μm). **b** Quantification of Iba1^+^ microglia in the hippocampus (*n* = 3, 3 images per mouse). **c-d** Characterization of microglia morphology via cell solidity (**c**) and circularity (**d**) (*n* = 3, 10–12 cells per mouse). Resting microglia are highly ramified while activated microglia present an amoeboid shape, with no or small ramifications. Activated microglia are characterized by a higher circularity and solidity. Con: control group; ConL: Con + Lentinan group; Tg: chronic *T. gondii*-infected group; TgL: Tg + Lentinan group. Values are mean ± SEM. *P < 0.05, **P < 0.01, ***P < 0.001

Hyperactivation of microglia has been considered a crucial cause of behavioral changes induced by *T. gondii* [54]. Here, we observed decreased transcript levels of genes related to microglial activation after lentinan treatment (Fig. 2f). Therefore, we were interested in whether lentinan could inhibit *T. gondii*-induced microglial activation. Using Iba1 as the marker of microglia, we observed an increased microglia cell number (Iba1^+^ cells) in the hippocampal CA1, CA3 and DG regions of *T. gondii*-infected mice (CA1: *F*_(3, 36)_ = 2.393, *P* < 0.001; CA3: *F*_(3, 36)_ = 0.9481, *P* < 0.001; DG: *F*_(3, 36)_ = 2.185, *P* < 0.001, Fig. 4a, b), which was reduced after lentinan treatment (CA1: *F*_(3, 36)_ = 2.393, *P* < 0.01; CA3: *F*_(3, 36)_ = 0.9481, *P* < 0.01; DG: *F*_(3, 36)_ = 2.185, *P* < 0.01, Fig. 4a, b). Sholl analysis of Iba1^+^ cells showed that the solidity index of microglia in the hippocampal CA1, CA3 and DG regions of *T. gondii*-infected mice was prominently increased compared with that of control mice. However, lentinan decreased the solidity index in these areas (CA1: *F*_(3, 36)_ = 1.255, *P* < 0.05; CA3: *F*_(3, 36)_ = 4.580, *P* < 0.01; DG: *F*_(3, 35)_ = 0.7123, *P* < 0.05, Fig. 4c). Similar results were found in the circularity index of microglia (CA1: *F*_(3, 36)_ = 1.113, *P* < 0.05; CA3: *F*_(3, 36)_ = 2.456, *P* < 0.01; DG: *F*_(3, 35)_ = 1.470, *P* < 0.01, Fig. 4d). Overall, these findings indicated that lentinan abated microglial activation in the hippocampus of *T. gondii*-infected mice.

### Lentinan inhibited the proinflammatory cytokines and C1q production initiated by *T. gondii* infection in the hippocampus

**Fig. 5:**
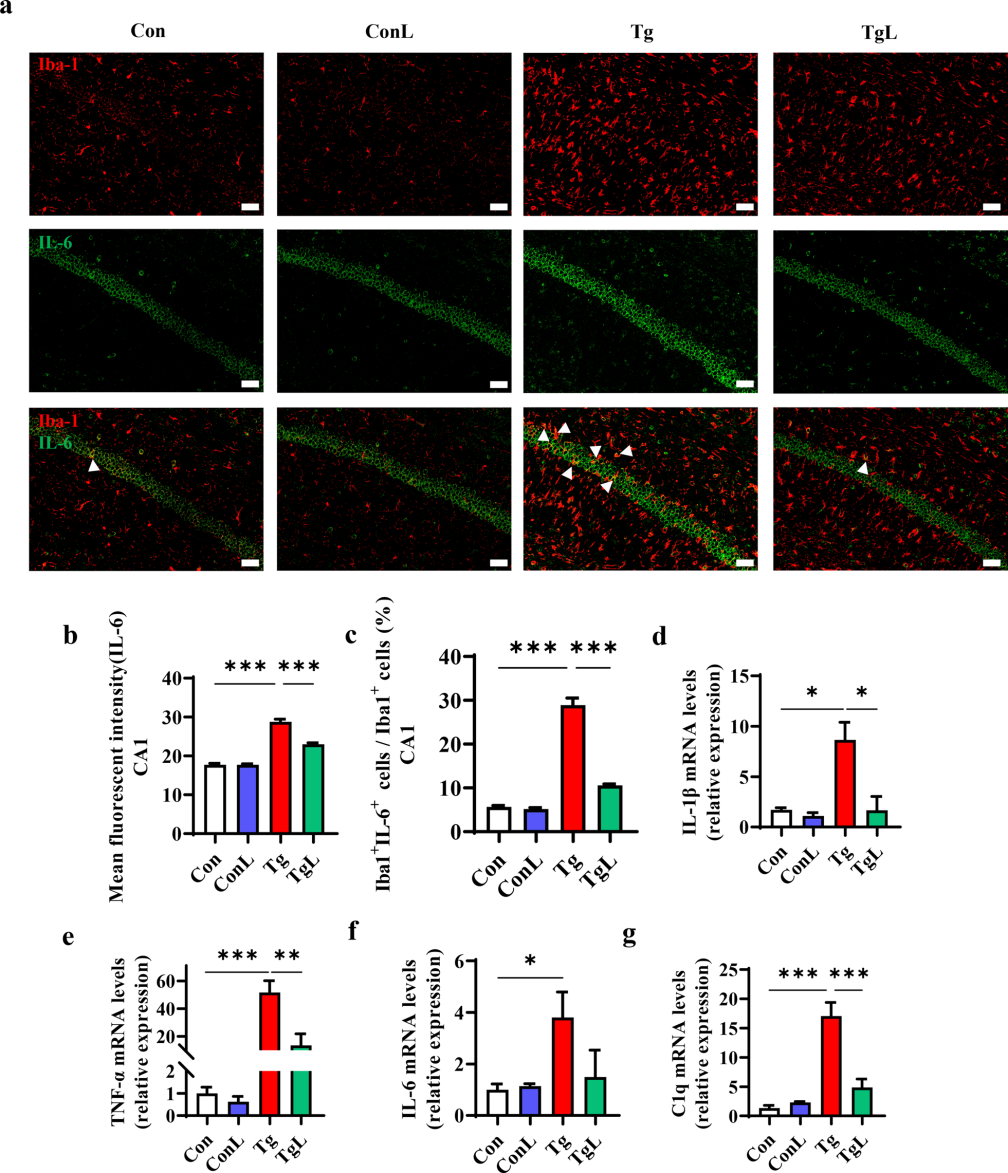
Lentinan inhibited the pro-inflammatory cytokines and C1q production initiated by *Toxoplasma gondii* infection in the hippocampus. **a** Double immunofluorescence staining for Iba1 (red) and IL-6 (green) in the CA1 region of the hippocampus of Con, ConL, Tg and TgL mice (Scale bar: 50 μm). **b** Quantification of the mean fluorescence intensity of IL-6^+^ cells in the CA1 region of the hippocampus (*n* = 3, 3 images per mouse). **c** Percentage of Iba1^+^IL-6^+^ cells in Iba1^+^ cells (*n* = 3, 3 images per mouse). **d–g** mRNA expression of IL-1β, TNF-α, C1q, and IL-6, (*n* = 6–8). Con: control group; ConL: Con + Lentinan group; Tg: chronic *T. gondii*-infected group; TgL: Tg + Lentinan group. Values are mean ± SEM. *P < 0.05, **P < 0.01, ***P < 0.001

After determining the effect of lentinan on microglial activation, we further characterized the profile of proinflammatory cytokines and complement components. We observed the increased immunofluorescence intensity of IL-6 and the increased number of IL-6-positive microglia in the hippocampal CA1, CA3 and DG of *T. gondii*-infected mice, although most IL-6 immunoreactivity was in the neuronal layer. However, this alteration was reversed after lentinan treatment (*F*(3, 36) = 1.894, *P* < 0.001, Fig. 5b; *F*(3, 36) = 6.684, *P* < 0.001, Fig. 5c; Additional file 1: Fig. S2). Consistently, lentinan significantly inhibited the upregulation of IL-1β and TNF-α mRNA levels in the hippocampus of the infected mice (*F*_(3, 9)_ = 2.149, *P* < 0.05, Fig. 5d; *F*_(3, 15)_ = 3.120, *P* < 0.01, Fig. 5e), although IL-6 mRNA levels were not affected (Fig. 5f). Furthermore, a previous study reported that complement Factor C1q was associated with microglial activation, *T. gondii* clearance and neuron damage [55]. We showed a significant increase in C1q mRNA levels in the hippocampus of the infected mice, while lentinan strikingly abrogated this increase (*F*_(3, 16)_ = 6.752, *P* < 0.001, Fig. 5g). Overall, these results suggested that lentinan prevented neuroinflammation and C1q production in the hippocampus postinfection.

### Lentinan mitigated neurite degeneration and increased the density and innervation of dendritic spines in the hippocampal CA1 region of *T. gondii*-infected mice

**Fig. 6:**
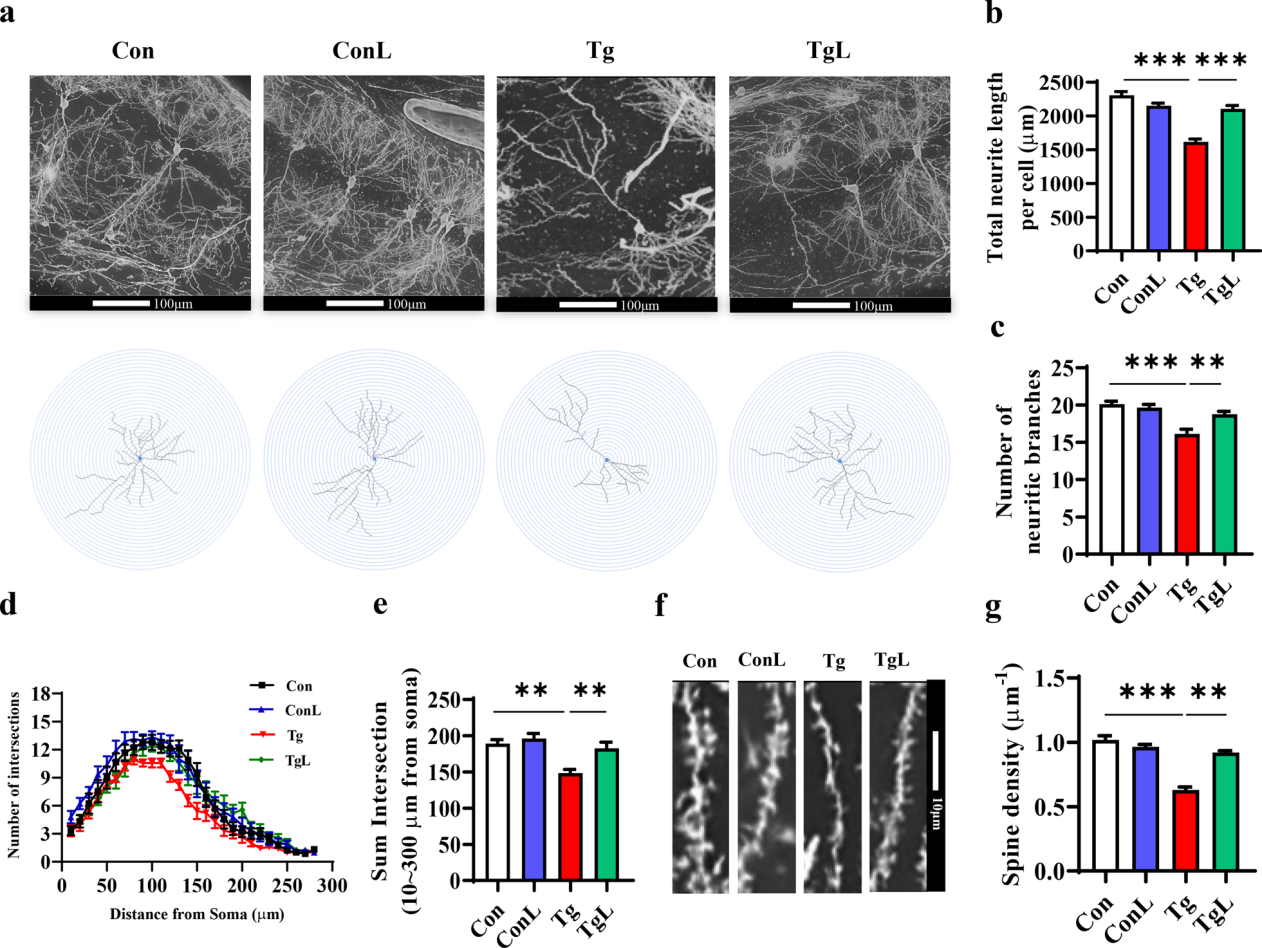
Lentinan mitigated neurite degeneration and increased dendritic spines in the hippocampal CA1 region of *Toxoplasma gondii*-infected mice. **a** Representative images of pyramidal neurons in the CA1 region of the hippocampus of control (Con), ConL, Tg and TgL mice (scale bar: 100 μm). **b** Total neurite length and (**c**) number of neuritic branches per cell (*n* = 18–20). **d** Sum intersection (10–300 μm from soma) per cell (*n* = 18–20). **e** Sholl analysis showing the number of intersections at different distances from soma (per 50 μm, 0–300 μm). **f**–**g** Representative images (scale bar: 10 μm) and quantification of dendritic spines of neurons in the CA1 region of the hippocampus (*n* = 50). Con: control group; ConL: Con + Lentinan group; Tg: chronic *T. gondii*-infected group; TgL: Tg + Lentinan group. Values are mean ± SEM. **P < 0.01, ***P < 0.001

We further examined whether neurite damage initiated by *T. gondii* infection could be altered by lentinan via Golgi-Cox staining. Representative images of hippocampal neurites are shown in Fig. 6a. We observed a shortened total neurite length per cell and a loss of neurite branches induced by *T. gondii* infection, while lentinan reversed these changes (*F*_(3, 32)_ = 0.6060, *P* < 0.001, Fig. 6b; *F*_(3, 32)_ = 1.144, *P* < 0.01, Fig. 6c). Moreover, Sholl analysis showed that lentinan mitigated the decreased number of dendritic intersections induced by *T. gondii* infection (Fig. 6d; *F*_(3, 32)_ = 1.225, *P* < 0.01, Fig. 6e). In addition, a lower spine density was observed in *T. gondii*-infected mice, which could be increased by lentinan (Fig. 6f; *F*_(3, 32)_ = 3.525, *P* < 0.01, Fig. 6g). These findings explain the beneficial effect of lentinan on neuronal complexity and dendritic spines.

### Lentinan improved synaptic ultrastructural impairment in the hippocampus of *T. gondii*-infected mice

**Fig. 7:**
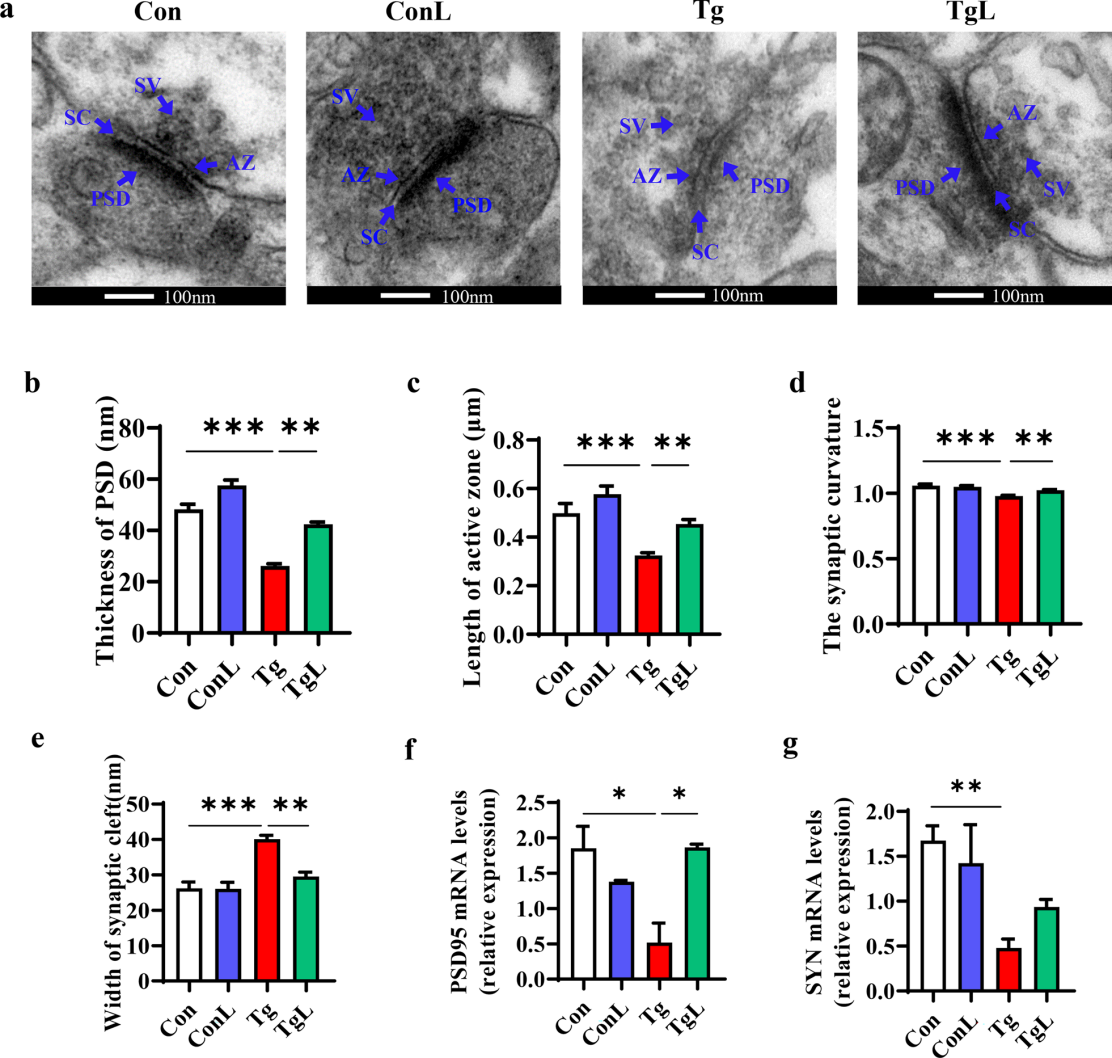
Lentinan improved the synaptic ultrastructural impairment in the hippocampus of *Toxoplasma gondii*-infected mice. **a** The ultrastructure of synapses on the electron micrograph in the hippocampus CA1 region of Con, ConL, Tg, TgL mice (scale bar: 100 nm). **b**–**e** Image analysis of the synaptic ultrastructure with software ImageJ (*n* = 3, 8 images per mouse): **b** Thickness of postsynaptic density. **c** Length of active zone. **d** The synaptic curvature. **e** Width of the synaptic cleft. **f** mRNA expression of postsynaptic density 95 (PSD95) in the hippocampus of Con, ConL, Tg, TgL mice (*n* = 6–8). **g** mRNA expression of synaptophysin (SYN) in the hippocampus of Con, ConL, Tg, TgL mice (*n* = 6–8). Con: control group; ConL: Con + Lentinan group; Tg: chronic *T. gondii*-infected group; TgL: Tg + Lentinan group. *SC* synaptic cleft, *SV* synaptic vesicle. Values are mean ± SEM. *P < 0.05, **P < 0.01, ***P < 0.001

The neuronal ultrastructure of synapses in the hippocampal CA1 region was examined with transmission electron microscopy (TEM). Representative images of hippocampal synaptic ultrastructure are shown in Fig. 7a. We observed a reduced thickness of the postsynaptic densities (PSD), shortened length of the active zone, decreased synaptic curvature and broadened synaptic cleft induced by *T. gondii* infection, while lentinan ameliorated these abnormities (*F*_(3, 36)_ = 3.167, *P* < 0.01, Fig. 7b; *F*_(3, 36)_ = 3.622, *P* < 0.01, Fig. 7c; *F*_(3, 36)_ = 2.678, *P* < 0.01, Fig. 7d; *F*_(3, 36)_ = 1.220, *P* < 0.01, Fig. 7e). Furthermore, we confirmed that postsynaptic density 95 (PSD95), an important postsynaptic protein for synapses, was significantly downregulated postinfection, while lentinan prevented this decline (*F*_(3, 6)_ = 1.297, *P* < 0.05, Fig. 7f). However, the downregulation of synaptophysin (SYN), an important presynaptic protein for synapses, was not reversed by lentinan. Generally, these results demonstrated that lentinan could ameliorate the damage to the hippocampal synaptic ultrastructure induced by chronic *T. gondii* infection.

### Lentinan inhibited the proliferation of *T. gondii* in BV2 cells in vitro

**Fig. 8:**
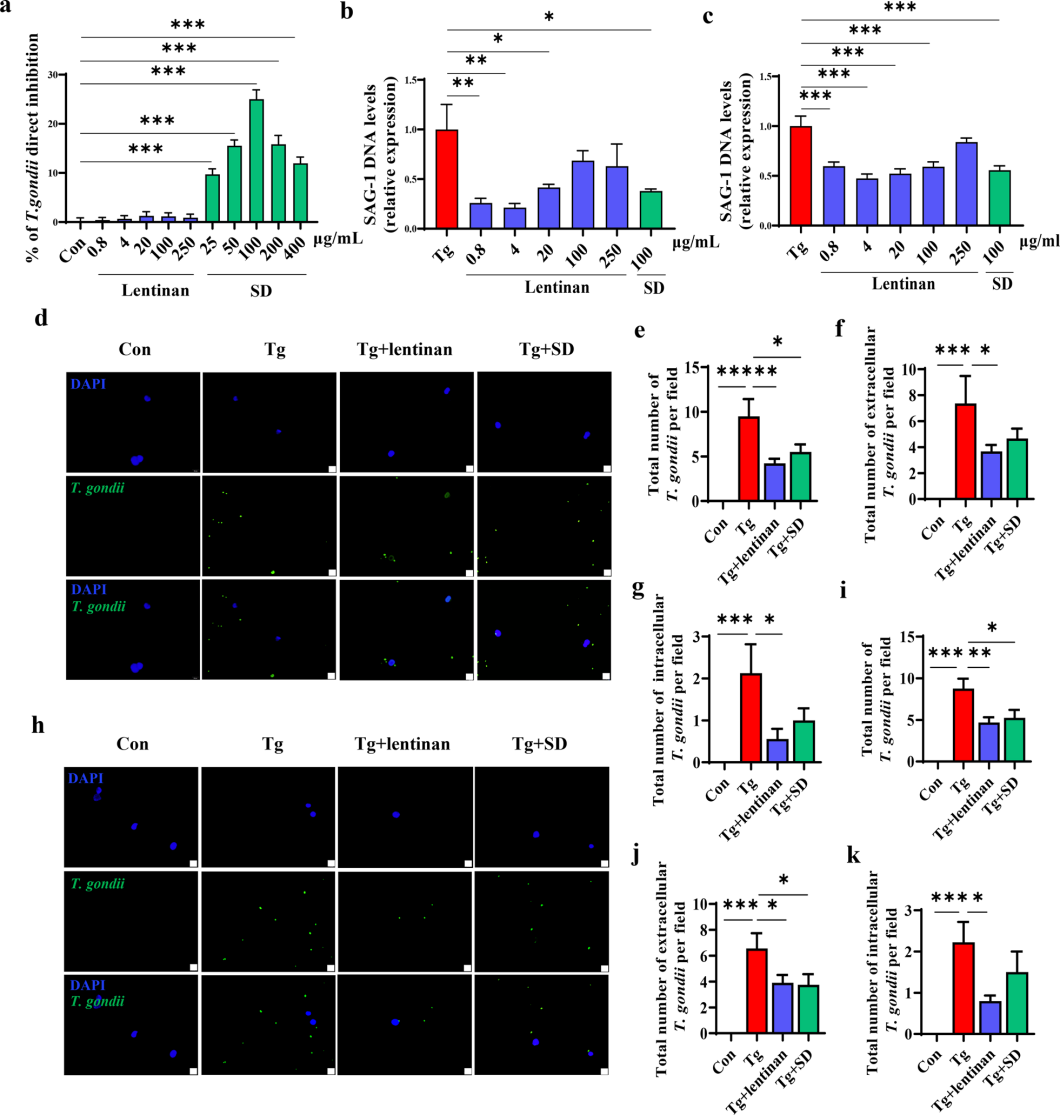
Lentinan inhibited the proliferation of *Toxoplasma gondii* in BV2 cells in vitro. *a* Direct inhibition rates on tachyzoites with indicated lentinan or SD treatment. **b** qRT-PCR analysis of SAG1 mRNA expression in BV2 cells for the prevention experiment. **c** qRT-PCR analysis for SAG1 mRNA expression in BV2 cells for the therapeutic experiment. **d**–**g** For the prevention experiment, immunofluorescence staining with anti-*T. gondii* antibody was performed to detect the effect of 4 μg/ml lentinan or 100 μg/ml SD on intracellular and extracellular *T. gondii* tachyzoites numbers in BV2 cells: **d** Representative immunofluorescence staining of *T. gondii* tachyzoites, the tachyzoites were indicated in green, whereas the DAPI stain (blue) indicated the location and size of nuclei (scale bar: 20 μm). **e** Total number of *T. gondii* per field. **f** Total number of extracellular *T. gondii* per field. **g** Total number of intracellular *T. gondii* per field. **h**–**k** For the therapeutic experiment, immunofluorescence staining with anti-*T. gondii* antibody was performed to detect the effect of 4 μg/ml lentinan or 100 μg/ml SD on intracellular and extracellular *T. gondii* tachyzoite numbers in BV2 cells: **h** representative immunofluorescence staining of *T. gondii* tachyzoites, the tachyzoites are indicated in green, whereas the DAPI stain (blue) indicates the location and size of nuclei (scale bar: 20 μm). **I** Total number of *T. gondii* per field. **J** Total number of extracellular *T. gondii* per field. **k** Total number of intracellular *T. gondii* per field. Con: control group; Tg: *T. gondii* tachyzoite-infected group; *SD* sulfadiazine. Values are mean ± SEM. *P < 0.05, **P < 0.01, ***P < 0.001

Since lentinan could decrease cyst burden in the brains of *T. gondii*-infected mice, we next determined whether lentinan has a direct anti-*T. gondii* effect or inhibits the proliferation of tachyzoites in BV2 cells according to a previously reported method [45, 46, 48, 49]. First, we cultivated free tachyzoites of the *T. gondii* RH strain with different concentrations of lentinan or SD (a classic anti-toxoplasmosis drug used in the clinic). We found that the inhibition rates on tachyzoites with 25, 50, 100, 200 and 400 μg/ml SD treatment differed significantly from those of the Con group, and the optimal inhibitory concentration of SD was 100 μg/ml (inhibition rate: 25.02 ± 1.89%, *F*_(10, 33)_ = 1.302, *P* < 0.001, Fig. 8a). However, the inhibition rates of 0.8, 4, 20, 100 and 250 μg/ml lentinan treatment were not significantly different from those of the Con group (Fig. 8a), indicating that lentinan cannot directly inhibit tachyzoite proliferation in vitro.

Next, we investigated the effects of lentinan or SD stimulation on *T. gondii*-infected BV2 cells. BV2 cells were infected with tachyzoites after or before exposure to lentinan or SD. We found that pretreatment with lentinan (0.8, 4 and 20 μg/ml) and 100 μg/ml SD could significantly decrease the mRNA levels of SAG1 in infected BV2 cells (*F*_(6, 19)_ = 0.6802, *P* < 0.05, Fig. 8b). Interestingly, these dose of lentinan and SD also had a therapeutic effect as evidenced by the downregulation of SAG1 mRNA in the treatment groups (*F*_(6, 21)_ = 1.326, *P* < 0.001, Fig. 8c). Correspondingly, using immunofluorescence staining with an anti-*T. gondii* antibody, we found that both the intracellular and extracellular numbers of tachyzoites were lower in the Tg + lentinan group than in the infected group in both prevention and treatment studies (*F*_(3, 31)_ = 5.607, *P* < 0.01, Fig. 8e; *F*_(3, 32)_ = 3.991, *P* < 0.05, Fig. 8f; *F*_(3, 32)_ = 4.276, *P* < 0.05, Fig. 8g; *F*_(3, 33)_ = 6.762, *P* < 0.01, Fig. 8i; *F*_(3, 33)_ = 3.503, *P* < 0.05, Fig. 8j; *F*_(3, 33)_ = 7.235, *P* < 0.05, Fig. 8k). Collectively, these results indicated that lentinan could inhibit the proliferation of tachyzoites in BV2 cells, which might contribute to the decreased cyst burden in the brains of infected mice.

## Discussion

The discovery of new effective drugs for chronic *T. gondii* infection-induced cognitive deficits is imperative yet challenging [10, 11]. Repositioning of existing drugs is a strategy to expedite drug development for toxoplasmosis [56]. Lentinan is used to treat tumors [26]. Previous studies have shown that lentinan can resist several protozoan infections [30, 31]. The present study investigated the effect of lentinan on cognitive deficits induced by *T. gondii* infection [57]. We observed that lentinan improved activities of daily living, spatial memory and recognition memory in mice. As one of the most successful parasites, *T. gondii* forms cysts filled with bradyzoites in the brain of hosts, which may remain in the brain permanently and affect host behavior [58, 59]. We found that the cyst number decreased after lentinan administration, which was correlated with the improvement of cognitive deficits. Furthermore, we showed that the antiparasitic effect of lentinan relied on microglia in vitro. In line with this study, previous studies have reported a potential neuroprotective effect of lentinan on cognitive deficits and anxiety in mice [33, 34]. Altogether, these results indicate that lentinan may be a drug candidate for preventing *T. gondii*-induced cognitive decline.

To illustrate the mechanism by which lentinan improves cognitive impairment, the hippocampal transcriptome profile was determined by RNA-seq. We found that pathways related to synaptic function (e.g. synaptic plasticity and synaptic transmission) and neural development were downregulated post infection. These findings were in line with those from another recent report [53]. However, lentinan treatment abrogated these transcriptome changes. Numerous studies have reported the disruption of neurite arborization and synaptic plasticity in neurodegenerative disorders [60, 61] and toxoplasmosis [4]. For example, *T. gondii* infection is reported to induce synaptic loss, impair neural circuits and damage the synaptic ultrastructure in mice [53, 62]. The present study also observed impairment of neuronal integrity and synaptic ultrastructure in *T. gondii*-infected mice, which was improved with lentinan treatment. The dysfunction of synaptic-related proteins is closely involved in the development of neuropsychosis [63, 64]. SYN, a calcium-binding glycoprotein located in the membrane of synaptic vesicles, regulates the differentiation and growth of axon dendrites and the structure of synapses [65]. PSD95 is an important cytoskeleton protein in the postsynaptic density, and its downregulated expression indicates the impairment of synaptic transmission [66]. Notably, several studies have shown that the downregulation of SYN and PSD95 expression in the hippocampus was positively related to the cognitive impairment of AD patients and toxoplasmosis [53, 67]. As expected, we observed a decrease in the mRNA levels of SYN and PSD95 after *T. gondii* infection, and lentinan prevented the reduction in PSD95 levels. In addition, a previous study reported that TgCtwh6 infection induces apoptosis in hippocampal neurons [57]. This study also identified the upregulated biological processes associated with apoptosis post infection, which could be reversed by lentinan. These findings suggest that lentinan could prevent the impairment of neuronal integrity and synaptic ultrastructure induced by chronic *T. gondii* infection, supporting the improvement of cognitive deficits.

As one of the innate immune cell types in the brain, microglia play an important role in pathogen elimination. A study has shown that activating the receptor of lentinan, Dectin-1, can enhance the phagocytosis of *Candida albicans* by retinal microglia in vitro [68]. Another study reported that Dectin-1 activation can promote the phagocytosis of *Lomentospora prolificans* by BV2 cells [69]. Here, we found that lentinan efficiently decreased both intracellular and extracellular *T. gondii* tachyzoite numbers in BV2 cells and reduced the cyst burden in the brains of *T. gondii*-infected mice. However, lentinan could not directly inhibit the proliferation of tachyzoites in vitro. Thus, lentinan may act as a neuroimmunomodulatory molecule contributing to the improvement of cognitive deficits. Furthermore, the persistent activation of microglia can induce neuroinflammation, resulting in neuronal apoptosis and synaptic dysfunction and eventually causing cognitive impairment [70]. Microglia can also induce synapse pruning via complement C1q, which is indicated in the pathogenesis of various brain disorders [71]. A previous study reported that C1q activation is one part of the immune response against cysts of *T. gondii* [55]. In this study, we observed that chronic *T. gondii* infection highly upregulated the expression of IL-1β, IL-6, TNF-α and C1q in the hippocampus of mice. Moreover, biological processes, such as positive regulation of interleukin-1 beta production, inflammatory response and positive regulation of NF-κB transcription factor activity, were significantly upregulated post infection. In addition, markers (TLR4, CD68, Tyrobp and Cx3cr1) associated with microglial activation were significantly upregulated post infection. However, lentinan remarkably reversed these alterations. Overall, it is speculated that lentinan prevents cognitive deficits by regulating neuroinflammation.

## Conclusions

Our study has shown that lentinan can improve cognitive deficits caused by chronic *T. gondii* infection. Mechanistically, lentinan decreases cyst burden and prevents neuroinflammation, neurite impairment and synaptic loss in infected mice. These data provide novel insight for treating *T. gondii*-related neurodegenerative diseases.

### Supplementary Information

**Additional file 1: Table S1.** The qRT-PCR primer sequences used in the study. **Figure S1.** Cyst burden in the brain of infected mice and their correlation with behavior performance. **a** Cyst number in the partial brain. **b**–**e** Pearson’s correlational analysis was used to assess the correlation between cyst numbers and behavior changes. *P < 0.05. **Table S2.** Top 30 genes in 2250 DEGs were upregulated by *Toxoplasma gondii* but were downregulated by lentinan. **Table S3.** Genes related to cognitive function in 241 DEGs were downregulated by *Toxoplasma gondii* but upregulated by lentinan. **Figure S2.** Lentinan downregulated the neuroinflammation in the hippocampus caused by chronic *Toxoplasma gondii* infection. **a** Double immunofluorescence staining for Iba1 (red) and IL-6 (green) in the CA3 region of hippocampus of Con, ConL, Tg and TgL mice (Scale bar: 50 μm). **b** Quantification of the mean fluorescence intensity of IL-6^+^ cells in the CA3 region of hippocampus (*n* = 3, 3 images per mouse). **c** Percentage of Iba1^+^IL-6^+^ cells in Iba1^+^ cells in the DG region of hippocampus (*n* = 3, 3 images per mouse). **d** Double immunofluorescence staining for Iba1 (red) and IL-6 (green) in the DG region of hippocampus of Con, ConL, Tg, and TgL mice (scale bar: 50 μm). **e** Quantification of the mean fluorescence intensity of IL-6^+^ cells in the DG region of hippocampus (*n* = 3, 3 images per mouse). **f** Percentage of Iba1^+^IL-6^+^ cells in Iba1^+^ cells in the DG region of hippocampus (*n* = 3, 3 images per mouse). ***P < 0.001.

## Data Availability

The sequencing data used in this study have been deposited in SRA database with accession number: https://www.ncbi.nlm.nih.gov/bioproject/PRJNA859430.

## Abbreviations

*T. gondii*: *Toxoplasma gondii*
*L. edodes*: *Lentinula edodes*
PD: Parkinson’s disease
AD: Alzheimer’s disease
TOM: Temporal order memory
NMDAR: *N*-methyl-d-aspartate receptor
CNS: Central nervous system
TNF-α: Tumor necrosis factor-α
IL-6: Interleukin-6
IL-1β: Interleukin-1β
*L. donovani*: *Leishmania donovani*
HIV: Human immunodeficiency virus
HFD: High-fat diet
PBS: Phosphate buffer saline
OL: Objection location test
NOR: Novel object recognition test
PDI: Place discrimination index
NODI: Novel object discrimination index
TEM: Transmission electron microscopy
SYN: Synaptophysin
PSD: Postsynapse
SC: Synaptic clefts
AC: Active zone
DEGs: Differential expression genes
GO: Gene ontology
KEGG: Kyoto Encyclopedia of Genes and Genomes
qRT-PCR: Quantitative real-time PCR
SEM: Standard error of the mean
TLR4: Toll-like receptor4
PLD4: Phospholipase D family, member 4
Cx3cr1: CX3C chemokine receptor1
Plcb1: Phosphoinositide phospholipase C-beta-1
Gnb5: Guanine nucleotide-binding protein subunit beta-5
GluR2: Glutamate receptor2
Ppp3ca: Protein phosphatase 3 catalytic subunit alpha
GABRD: Gamma-aminobutyric acid receptor subunit delta
GABRA2: Gamma-aminobutyric acid receptor subunit alpha-2
HTR1B: 5-Hydroxytryptamine receptor 1B
