# Supplementary material for: Lentinan has a beneficial effect on cognitive deficits induced by chronic Toxoplasma gondii infection in mice
Source: Parasit Vectors. 2023 Dec 13;16:454. doi: 10.1186/s13071-023-06023-5 (PMC10717010; doi:10.1186/s13071-023-06023-5)
Supplement: Supplementary file 1 — Additional file 1: Table S1. The qRT-PCR primer sequences used in the study. Figure S1. Cyst burden in the brain of infected mice and their correlation with behavior performance. a Cyst number in the partial brain. b–e Pearson’s correlational analysis was used to assess the correlation between cyst numbers and behavior changes. *P < 0.05. Table S2. Top 30 genes in 2250 DEGs were upregulated by Toxoplasma gondii but were downregulated by lentinan. Table S3. Genes related to cognitive function in 241 DEGs were downregulated by Toxoplasma gondii but upregulated by lentinan. Figure S2. Lentinan downregulated the neuroinflammation in the hippocampus caused by chronic Toxoplasma gondii infection. a Double immunofluorescence staining for Iba1 (red) and IL-6 (green) in the CA3 region of hippocampus of Con, ConL, Tg and TgL mice (Scale bar: 50 μm). b Quantification of the mean fluorescence intensity of IL-6+ cells in the CA3 region of hippocampus (n = 3, 3 images per mouse). c Percentage of Iba1+IL-6+ cells in Iba1+ cells in the DG region of hippocampus (n = 3, 3 images per mouse). d Double immunofluorescence staining for Iba1 (red) and IL-6 (green) in the DG region of hippocampus of Con, ConL, Tg, and TgL mice (scale bar: 50 μm). e Quantification of the mean fluorescence intensity of IL-6+ cells in the DG region of hippocampus (n = 3, 3 images per mouse). f Percentage of Iba1+IL-6+ cells in Iba1+ cells in the DG region of hippocampus (n = 3, 3 images per mouse). ***P < 0.001. [file 13071_2023_6023_MOESM1_ESM.docx]

**Additional file 1**

**Table S1.** The RT-PCR primer sequences used in the study.

| **Mouse Gene** | **Forward Primer (5’→3’)** | **Reverse Primer (5’→3’)** |
| --- | --- | --- |
| β-actin | AGAAGGTGGTGAAGCAGGCATC | CGAAGGTGGAAGAGTGGGAGTTG |
| C1q | TTCGGCAGAACCCAATGACG | TGGTATGGACTCTCCTGGTTG |
| IL-1β | TGGGAAACAACAGTGGTCAGG | CTGCTCATTCACGAAAAGGGA |
| TNF-α | CTTGTTGCCTCCTCTTTTGCTTA | CTTTATTTCTCTCAATGACCCGTAG |
| IL-6 | TCACAGAAGGAGTGGCTAAGGACC | ACGCACTAGGTTTGCCGAGTAGAT |
| PSD95 | TCCGGGAGGTGACCCATTC | TTTCCGGCGCATGACGTAG |
| SYN  SAG1 | CGCACCTCGGACAAGTCTC  GTCGTTCTTGCGATGTGG | CCCGAAGGCGAAAATAGCAAA  TTTGCCTGTTGGGTGAGTA |

 ****

**e**

**d**

**c**

**b**

**a**

**Figure. S1 Cyst burden in the brain of infected mice and their correlation with behaviour performance. a** Cyst number in the partial brain. **b-e** Pearson’s correlational analysis was used to assess the correlation between cyst numbers and behavior changes. **P* < 0.05.

**Table S2.** Top 30 genes in 2250 DEGs were upregulated by *T. gondii*, while were downregulated by lentinan.

**Table S3.** Genes related to cognitive function in 241 DEGs were downregulated by *T. gondii,* while were upregulated by lentinan.

**a**

**d**

**f**

**e**

**c**

**b**


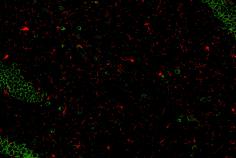

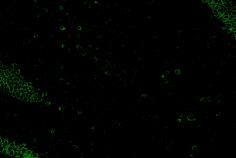

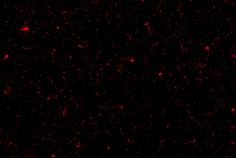

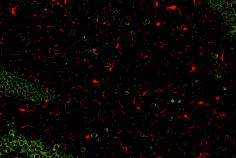

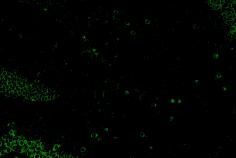

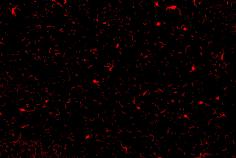


**Iba-1**

**IL-6**

**IL-6**

**Iba-1**

**Con**

**ConL**

**Tg**

**TgL**


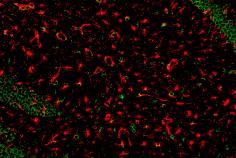

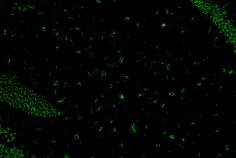

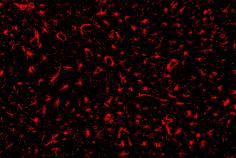

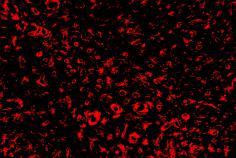

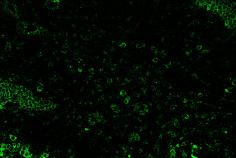

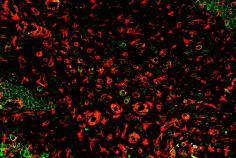

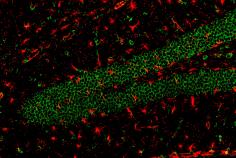

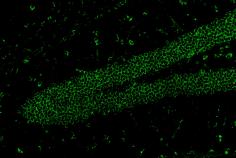

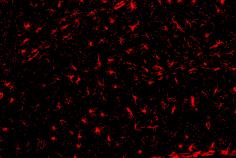

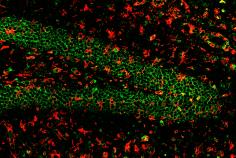

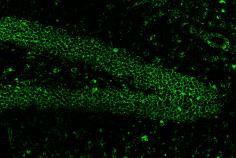

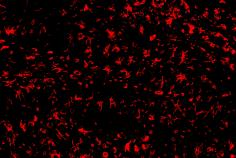

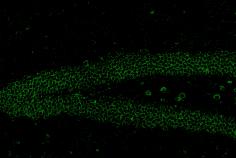

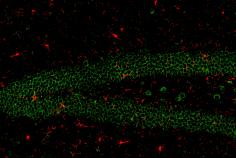

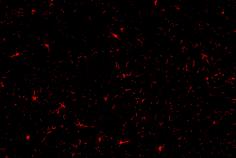

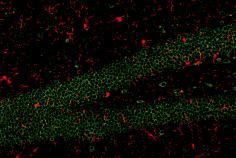

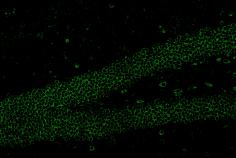

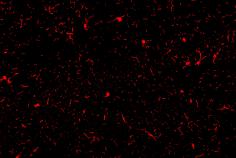


**Iba-1**

**IL-6**

**IL-6**

**Iba-1**

**Con**

**ConL**

**Tg**

**TgL**

**Figure S2. Lentinan downregulated the neuroinflammation in the hippocampus induced by chronic *T. gondii* infection. a** Double immunofluorescence staining for Iba1 (red) and IL-6 (green) in the CA3 region of hippocampus of Con, ConL, Tg, and TgL mice (Scale bar: 50 μm). **b** Quantification of the mean fluorescence intensity of IL-6^+^ cells in the CA3 region of hippocampus (n = 3, 3 images per mouse). **c** Percentage of Iba1^+^IL-6^+^ cells in Iba1^+^ cells in the DG region of hippocampus (n = 3, 3 images per mouse). **d** Double immunofluorescence staining for Iba1 (red) and IL-6 (green) in the DG region of hippocampus of Con, ConL, Tg, and TgL mice (Scale bar: 50 μm ). **e** Quantification of the mean fluorescence intensity of IL-6^+^ cells in the DG region of hippocampus (n = 3, 3 images per mouse). **f** Percentage of Iba1^+^IL-6^+^ cells in Iba1^+^ cells in the DG region of hippocampus (n = 3, 3 images per mouse). ****P* < 0.001.
